# Tramesan, a novel polysaccharide from *Trametes versicolor*. Structural characterization and biological effects

**DOI:** 10.1371/journal.pone.0171412

**Published:** 2017-08-22

**Authors:** Marzia Scarpari, Massimo Reverberi, Alessia Parroni, Valeria Scala, Corrado Fanelli, Chiara Pietricola, Slaven Zjalic, Vittoria Maresca, Agostino Tafuri, Maria R. Ricciardi, Roberto Licchetta, Simone Mirabilii, Aris Sveronis, Paola Cescutti, Roberto Rizzo

**Affiliations:** 1 Sapienza University, Dept. of Environmental Biology, P.le Aldo Moro 5, Roma, Italy; 2 Research Unit for Plant Pathology, Council for Agricultural Research and Economics, Rome, Italy, Roma, Italy; 3 Department of Ecology, Agronomy and Aquaculture, University of Zadar, HR, Zadar; 4 IFO-S- Gallicano, Roma, Italy; 5 Department of Clinical and Molecular Medicine, Hematology, "Sant'Andrea" University Hospital Sapienza, University of Rome Roma; 6 Dept Life Sciences, Univ. Trieste, Trieste, Italy; Universita degli Studi di Pisa, ITALY

## Abstract

Mushrooms represent a formidable source of bioactive compounds. Some of these may be considered as biological response modifiers; these include compounds with a specific biological function: antibiotics (e.g. plectasin), immune system stimulator (e,g, lentinan), antitumor agents (e.g. krestin, PSK) and hypolipidemic agents (e.g. lovastatin) *inter alia*. In this study, we focused on the Chinese medicinal mushroom “yun zhi”, *Trametes versicolor*, traditionally used for (cit.) “*replenish essence and qi (vital energy)*”. Previous studies indicated the potential activity of extracts from culture filtrate of asexual mycelia of *T*. *versicolor* in controlling the growth and secondary metabolism (e.g. mycotoxins) of plant pathogenic fungi. The quest of active principles produced by *T*. *versicolor*, allowed us characterising an exo-polysaccharide released in its culture filtrate and naming it Tramesan. Herein we evaluate the biological activity of Tramesan in different organisms: plants, mammals and plant pathogenic fungi. We suggest that the bioactivity of Tramesan relies mostly on its ability to act as *pro* antioxidant molecule regardless the biological system on which it was applied.

## Introduction

Polysaccharides alone or conjugated to proteins are biopolymers exhibiting a variety of biological functions; from a structural point of view, this is due to the huge amount of different chemical and conformational motifs they can possess using different i) sugar composition, ii) glycosidic linkages and iii) branching. Actually, only 8x10^3^ tripeptides arise using 20 amino acids, but 6x10^6^ structurally different trisaccharides can be arranged using 20 different monosaccharides. Cells have evolved receptors with particular domains for carbohydrates [1] and polysaccharides can be recognized as such or as glycoproteins moieties. Indeed, the density and number of glycan epitopes can modulate the ligand/receptor interactions [2] and ultimately drive the fate of host-pathogen challenge [3].

In host-fungal interactions, glucans, cell wall-bound as well as secreted ones, are gaining momentum within scientific community since the incidence of fungal infections has been steadily rising in the past decades, in plant crops as well as in humans [4,5]. Among various polysaccharides, β-glucans of different sizes and branching patterns display significant and variable immune potency [6] and a significant number of studies on fungal β-glucans and their host receptors (such as Toll Like Receptors, TLR, and Dectin-1) have been performed [6].

Most fungal pathogens (e.g. *Candida albicans*, *Aspergillus fumigatus*) as well as non-toxic (edible) fungi (e.g. *Lentinula edodes*) display a species-specific pattern of cell wall glycans [7]. Two groups of glucans are present in fungi, α- and β-glucans. Some fungi have α-1,4-linked glucose units in their cell walls either to interconnect the linear α-1,3-glucan chains or in an alternated way with α-1,3-linked glucose units (e.g. nigeran in *Aspergillus niger*) [8]. The most abundant β-glucan in the fungal cell wall is a β-1,3-glucan which account for between 65% and 90% of the whole β-glucan content [9] together with the cell wall linked water insoluble β-1,4-linked poly-N-acetyl-D-glucosamine (chitin) [10]. These glucans may act as molecular/pathogen associated molecular patterns (MAMP or PAMP). Hosts have evolved an array of pattern recognition receptors (PRRs) able to sense the different molecular moieties that compose fungal cell walls. PRRs for fungal PAMPs include Toll-like receptors (TLRs), C-type lectin receptors (CLRs) and galectin family proteins [5,10,11].

Although poorly studied, glycans are important in cell-to-cell communication even in fungi. The most studied issue within fungus/fungus interactions is related to self/non-self-recognition between vegetative mycelia. This mechanism relies on *het* gene complex and ultimately leads fungus to programmed cell death under vegetative incompatibility circumstances [12,13]. Notably, TOL receptor in *Neurospora crassa* possesses a LRR domain, similar to plant and humans PRR, which allows fungus to distinguish self from non-self [12].

Host cell may react similarly towards fungal glucans. After having recognised them as non-self/MAMP, host cells activate defence strategies such as the cell antioxidant system [5,12]. Examples may be provided for mammals, plants and fungi. In mammals, dectin-1, assisted by TLRs, recognizes fungal ß-glucans and triggers ROS formation and activation of the master regulator of the antioxidant cellular defence Nrf-2. The downstream pathways may lead to cell death or antimicrobial immunity [14,15]. As an example in plants, Cerk1, together with CEBiP, recognizes fungal chitin and activates defence responses [16,17]. Fungi induce formation of ROS with a consequent alteration of cell redox balance. Recently, the close link between ROS perception and defence activation was described for the tomato WRKY transcriptional factor SlDRW1 required for disease resistance against *Botrytis cinerea* and tolerance to oxidative stress [18]. In fungi, the semi-purified fraction of lentinan, a ß-glucan of the basidiomycete *Lentinula edodes*, modulates oxidant/antioxidant balance in *Aspergillus* sect. Flavi by manipulating the expression of the oxidative stress-related transcription factor Ap*yapA* [19,20]. In turn, several features of *A*. *parasiticus* are modified by recognition of lentinan: morphology, growth and secondary metabolism [19]. The lectins recognition is required for coiling around the prey mycelium and formation of helix shaped hyphae in mycoparasitism. In *Hypocrea virens* the sucrose transporter is induced in the early stages of root colonization [21]. Thus, fungal glucans acting as elicitors may trigger several responses in different hosts. Amongst these, fungal polysaccharides may elicit the activation of cell redox balancers such as the oxidative stress-related transcription factors Yap-1 whose mechanism of action was firstly described in *Saccharomyces cerevisiae* [20].

Despite the increasing biological relevance of fungal polysaccharides, the majority of glucans investigated are derived from fungal extracts [6] obtained applying poor purification procedures which hinder result standardization and render the characterization of their biological activity, confused and, in some cases, contradictory.

In this study, we describe the identification of a biological active saccharidic fraction in the filtrate of the medicinal mushroom *T*. *versicolor* and the partial chemical characterization of the polysaccharide therein contained (henceforth referred as **Tramesan**). Its ability to trigger an antioxidant response in different biological systems (human cell lines, plants, and fungi) is reported.

## Materials and methods

### Fungal strain and growth conditions

*Trametes versicolor* strain C used in this study was registered at CABI biosciences (UK) and deposited in the culture collection of Department of Environmental Biology of Sapienza University of Rome as ITEM 117. *T*. *versicolor* C was grown for 7 days in potato dextrose broth (PDB, Himedia) and incubated at 25°C under shaken conditions (100 rpm). The liquid culture was homogenized, in sterile condition in Waring blender 8012. After homogenization, an aliquot (5% v/v) of the fungal culture was inoculated in 500 mL of PDB in 1L-Erlenmeyer flasks and incubated for 14 days at 25°C under rotary shaken conditions (100 rpm). The mycelia were then separated from the culture filtrates by subsequent filtrations with different size filters (Whatmann) to eliminate all the mycelia. Mycelia-purified culture filtrate was lyophilized and utilized for subsequent analyses. The isolates were kept in Potato Dextrose Agar at 4°C and the cultures were sub-cultured every 30 days.

### Purification of polysaccharide fractions

A suspension of the lyophilized *T*. *versicolor* culture filtrate (1g in 30 mL of H_2_O) was filtered to separate insoluble from soluble part. The remaining solution was cooled and precipitated with 4 volumes of cold ethanol. The precipitate was recovered by centrifugation at 1200 x*g* (20 min at 4°C), dissolved in 8 mL of 10 mM phosphate buffer pH 7.5 and treated with pronase E (Sigma Aldrich) at 37°C for 16 hr. After dialysis (12000 Da membrane Cut Off), the sample was recovered by lyophilisation (yield = 60 mg/L).

The polysaccharide fraction was separated by low-pressure size exclusion chromatography on a Sephacryl S-300 column (fractionation domain: 1–400 kDa for dextrans; gel bed volume: 1.6 id x 90 cm), using 50 mM NaNO_3_ as eluent at a flow rate of 6 mL/h. About 30 mg of the sample was dissolved in 1.9 mL of eluent and centrifuged before being loaded onto the column. Fractions of 2 mL were collected at 20 min intervals. Elution was monitored using a refractive index detector (WGE Dr. Bures, LabService Analytica), connected to a paper recorder and interfaced with a computer via PicoLog software. Polysaccharide molecular mass (MM) was evaluated by high performance size exclusion chromatography (HP-SEC) on an Agilent Technologies 1200 series HPLC equipped with three columns in series (Tosoh Bioscience, TSKgel G3000PW, G5000PW and G6000PW, i.d. 7.5 mm, length 30 cm) kept at 40°C with a thermostat (Waters Millipore). Calibration of the chromatographic system was performed using pullulan standards exhibiting molecular masses in the range 1.66x10^6^–5.90x10^3^ (Polymer Laboratories, Germany and Sigma for pullulan with MM = 1.66x10^6^). The calibration curve is reported in Figure A in S1 File. Elution was performed with 0.15 M NaCl, with a flow rate of 0.5 mL/min and monitored using a refractive index detector (Knauer, Labservice Analytica), interfaced with a computer via Agilent software.

### Composition analysis of Tramesan

In order to determine the composition in terms of neutral monosaccharides, the polysaccharide fraction was hydrolysed with 2 M trifluoroacetic acid (TFA) at 125°C for 1 h and the monosaccharides obtained were derivatised to alditol acetates [22]. The products were separated by gas–liquid chromatography (GLC) on a Perkin-Elmer Autosystem XL gas chromatograph equipped with a flame ionisation detector, an SP2330 capillary column (Supelco, 30 m), using *He* as carrier gas. The temperature program applied was 200–245°C at 4°C/min. Identification of the monosaccharides in Tramesan was achieved by comparing the retention times of each peak with those of standard sugars, previously derivatized in the same way. Quantification was obtained using inositol as internal standard.

### Linkage analysis of Tramesan

The glycosidic linkage position for each sugar residue was achieved by methylation analysis. The polysaccharide was permethylated according to the protocol by Harris [23], hydrolysed with 2 M TFA at 125°C for 1 h, and the products were then derivatized to alditol acetates [22], thus obtaining a mixture of partially methylated alditol acetates (PMAA). The alditols are substituted with a methyl group on the hydroxyls groups that were originally not engaged in linkages while acetyl esters are formed on the hydroxyl functions that were originally engaged in glycosidic linkages and in ring formation. The elution time in the GLC chromatogram led to the identification of the sugar type, and integration of the peak areas, after correcting by the effective carbon response factors [24], gave quantitative information. GLC-MS mass spectra identified the ring size and the position of glycosidic bonds. The PMAA were analysed by GLC using the same instrument and settings as for the composition analyses but with the temperature program: 150–250°C at 4°C/min. GLC-MS analyses were carried out on an Agilent Technologies 7890A gas chromatograph coupled to an Agilent Technologies 5975C VL MSD.

### Partial hydrolysis of Tramesan and characterisation of the products

Since the glycosidic linkages of 6-deoxy-sugars are more labile compared to those engaged by other monosaccharides, the polysaccharide was subjected to mild acid treatment in order to produce oligosaccharides that are suitable to electro-spray ionization mass spectrometry (ESI-MS). The mass spectra gave information on the monosaccharide composition and fragmentation of the parent ions revealed the position of the 6-deoxy sugars thanks to the different molecular mass of 6-deoxy-sugars and hexoses. 2 mg of Tramesan were treated with 0.5 M TFA at 100°C for 2 h. The hydrolysate was dried under a N_2_ flow, dissolved in 50% aqueous methanol—11 mM NH_4_OAc and subjected to ESI-MS experiments using a Bruker Esquire 4000 ion trap mass spectrometer connected to a syringe pump for the injection of the samples. The instrument was calibrated using a tune mixture provided by Bruker. In order to increase the detection sensitivity, oligosaccharides were reduced with NaBH_4_ [25], to label the reducing end and subsequently permethylated [26], dissolved in a 1:1 chloroform: methanol mixture, 11mM NH_4_OAc and subjected to ESI-MS and MS^2^ analyses. Samples were injected at 180 μL/h and detection was performed in the positive ion mode. After having verified that the hydrolysis conditions produced the desired oligosaccharides, the partial hydrolysis reaction was repeated on 10 mg of Tramesan, the hydrolysate was rotovaporated to dryness under reduced pressure at 45°C to eliminate residual TFA, taken to pH = 7.2, rotovaporated to dryness again, dissolved in 1.9 mL of 50 mM NaNO_3_, centrifuged and separated by size exclusion chromatography on a Bio Gel P2 column (fractionation domain: 100–1800 Da; gel bed volume: 1.6 cm i.d. × 90 cm), using 50 mM NaNO_3_ as eluent at a flow rate of 6 mL/h and the same system set up reported above. Fractions of 1.5 mL were collected at 15 min intervals and those belonging to the same peak were pooled together, and desalted on a Bioline preparative chromatographic system equipped with a Superdex G30 column (fractionation domain: up to 10 kDa; gel bed volume: 90 cm x 1.0 cm i.d., flow rate 1.5 mL/min) previously equilibrated in H_2_O. Elution of each peak was monitored with a Refractive Index detector (Knauer, LabService Analytica). The purified oligosaccharides were analysed by NMR spectroscopy.

### NMR spectroscopy

When necessary, polysaccharide fractions were de-O acetylated with 10 mM NaOH at room temperature for 5 h, under N_2_ flow. Samples were exchanged two times with 99.9% D_2_O by lyophilisation and then dissolved in 0.6 mL of 99.96% D_2_O. Spectra were recorded on a 500 MHz VARIAN spectrometer operating at 50°C for polysaccharides solution and at 25°C for oligosaccharide solution. 2D experiments were performed using standard VARIAN pulse sequences and pulsed field gradients for coherence selection when appropriate. HSQC spectra were recorded using 140 Hz (for directly attached ^1^H–^13^C correlations). TOCSY spectra were acquired using 120 ms spin-lock time and 1.2 s relaxation time. NOESY experiments were recorded with 200 ms mixing time and 1.5 s relaxation time. Chemical shifts are expressed in ppm using acetone as internal reference (2.225 ppm for ^1^H and 31.07 ppm for ^13^C). NMR spectra were processed using MestreNova software.

### Assay of Tramesan in fungi

*A*. *flavus* (Speare) *NRRL 3357and A*. *parasiticus* (Speare) NRRL 2999, both producers of aflatoxin B1 (AFL B1), were grown on PDA at 30°C for 7 days and from these cultures a suspension of 100 conidia, of each strain, independently, in 10 μL of sterilised distilled water was inoculated in 190 μL of PDB in presence or absence of Tramesan 0.38 μM, using 96-wells microplates. The cultures were incubated at 30°C for 3 days. The assay allowed us to test all the fractions in minimal amount and to generate hundreds of replications in a very short time (aflatoxin microtiter-based bioassay). Different cultures were independently filtrated with Millipore filters (0.22 μm). Then, the mycelia were lyophilized and weighted. The aflatoxin B1 was extracted adding, for each condition (control and treated with 0.19 and 0.38 μM of Tramesan), chloroform/methanol (2:1, v/v). The mixture was vortexed for 1 min, centrifuged and then the lower phases was drawn off. The extraction was repeated twice and the samples were concentrated under a N_2_ stream, re-dissolved in 50 μL of acetonitrile/water/acetic acid (20:79:1 v/v) and quantified by triple quad LC/MS 6420 (Agilent) with a method reported by Sulyok et al. [27], with minor modifications. Such modifications regarded mainly the use of Mycospin (Romer Labs) for the cleaning up of the samples prior analysis. The amount of aflatoxin B1 was evaluated by using an ISTD-normalised method in MassHunter workstation software, quantitative analysis version B.07.00. Aflatoxin B1-13C-d3 (Clearsynth) at 2 μM final concentration was used as ISTD. Aflatoxin B1 amount was expressed in ppb.

For the gene expression evaluation, total RNA from the mycelia of *A*. *flavus* and *A*. *parasiticus* strains was extracted, as reported by Scala et al. [28], 7 days after inoculation in PDB cultures amended or not (control) with Tramesan 0.38 μM and used to develop reverse-transcriptase quantitative PCR (RT-qPCR) assays for Af*yapA* (XM_002382086.1), Ap*yapA* (DQ104418.2), (*yapA primers*:; for 5’- GGTTGTTTGAGCCGTTGAGT -3’; rev 5’- ACGGCCTCAATAACAACGAC -3’), *sod1* (AFLA_099000; for 5’- AGTCGGTAAGGCAAACTGGG -3’; rev 5’- GAATTCGCCAGGACCAGACA -3’). RT-PCR were performed using SensiFAST™ SYBR® No-ROX™ (Bioline, Italy) at 95°C for 2 min; 30 cycles of 95°C for 15 s, 54–58°C (according to primer selected) for 30 s and 72°C for 15 s. The specificity of the reaction was verified by melt curve analysis and the efficiency of each primer was checked using the standard curve method. Primers with slopes between −3.1 and −3.6, and reaction efficiencies between 90 and 110% were selected for the analysis. Gene expression in the fungal strains was calculated by using the 2^-ΔΔCt^ method, i.e. by normalizing transcript levels of the gene of interest (GOI) onto the transcript of a housekeeping gene β-tubulin for *A*. *flavus* (HF937107.1; for 5’- GCTGGAGCGTATGAACGTCT-3’; rev 5’- GTACCAGGCAGAACGAGGAC-3’) and *A*. *parasiticus* (L49386.1; for 5’- TCACCTGCTCTGCCATCTTG-3’; rev 5’- TGTTGTTGGGGATCCACTCG-3’) as reported in Reverberi *et al*., 2011 [29] and onto their value in the untreated control (no Tramesan added). The housekeeping gene β-tubulin proved as being the most stable after analysis with the Normfinder algorithm (https://moma.dk/normfinder-software). The software for relative expression quantification provided with the Line GeneK thermocycler (Bioer, PRC) was used.

### Assay of Tramesan *in planta*

*Parastagonospora nodorum* (Berk.) was isolated from naturally infected durum wheat leaves (cv. Ciccio) cultivated in Italy. The strain ITEM 17131 was registered and conserved in the Culture Collection Agro Food Important Toxigenic Fungi-Item, Institute of Science of Food Production (ISPA), National Research Council (CNR), Via Amendola, 122/O, 70126 Bari, Italy (http://server.ispa.cnr.it/ITEM/Collection/). For in planta analysis, a growth in phytotron was used. Temperature, humidity and light were regulated in the chamber and notably, T = 20°C; humidity: 80%; light: 18 h of light with 150 μmol of photon/m^2^s, from 6.00 am to 22.00 pm). The plots were positioned in a rotary floor, so every plants were submitted to same conditions. Only a susceptible durum wheat variety (Svevo) was used in these tests. Kernels were disinfected by sodium hypochlorite (1%) during 10 min with permanent agitation of 150 rpm, and then rinsed three times with sterile distilled water during 5 min with permanent agitation of 150 rpm. The kernels germinated in vitro in water medium (0.5%). After, they were incubated at 20°C in dark during 24 h; 4°C in dark during 48 h; 20°C in dark during 24 h. The kernels were transferred in a two time autoclaved (20 min at 121°C) soil mixture (20 L of soil / 5 L of perlite), in pots of 0.5 L. The plants were irrigated three time a week, twice with 1 L of osmotic water. Plant leaves were sprayed with a solution of Tramesan 0.38 μM (100 mL per 64 plots) 48 hours prior pathogen inoculation. This latter occurred by spraying a picnidiospore suspension of *P*. *nodorum* on wheat second “real” leaf after flag leaf emergence (BBCH39). Visual identification of the disease and microscopic identification of *P*. *nodorum* pathogen were performed. In the phytotron, the SNB infection was visually assessed every 7–10 days post inoculation (dpi) as disease severity on flag leaf and as severity and incidence of the disease on ear using the Liu’ scale [30]. To assess fungal growth, we calculated fungal DNA present into plant tissues. Total DNA extracted from wheat leaves and seeds according to Färber method with minor modifications [19] and used for developing a specific SYBR green qPCR method by designing primers (for_ TGGGTACGCTTTTGAT CTCC; rev_ AACGAGGTGGTTCAGGTCAC) in the β-tubulin of *P*. *nodorum* (NCBI Gene Bank Ac. No. AY786332) as reported by Iori *et al*. [31]. For gene expression analysis, aliquots of 25 mg of lyophilized wheat leaves were powdered in liquid nitrogen and treated for RNA extraction. RNA extraction was performed with the TRI REAGENT method (Sigma-Aldrich, USA) and following manufacturer’ instructions and cDNA obtained using first Strand cDNA synthesis SUPER SCRIPT II for RT-PCR (Invitrogen, USA) kit. Real-time PCR was performed as described by Nobili *et al*. [32], using SensiFAST™ SYBR® No-ROX ™ (Bioline, Italy) at 95°C for 2 min; 30 cycles of 95°C for 15 s, 54–58°C (according to primer selected) for 30 s and 72°C for 15 s. The specificity of the reaction was verified by melt curve analysis and the efficiency of each primer was checked using the standard curve method. Primers with slopes between −3.1 and −3.6, and reaction efficiencies between 90 and 110% were selected for the analysis. The housekeeping gene β-tubulin of *T*. *turgidum* susp. *durum* (AJ971820.1; for 5’- GCTGCTGTATTGCAGTTGGC-3’; rev 5’- AAGGAATCCCTGCAGACCAG-3’), proved as being the most stable after analysis with the Normfinder algorithm (https://moma.dk/normfinder-software), was used as a reference for data normalization. The relative expression, as 2^− ΔΔ Ct^ values, of *PR9* wheat gene *PR9* (EU264058.1; for 5’-CAAGGTGAACTCGTGATGGA-3’; rev 5’-TTGAGGATTCAACCGTCGTT-3’), was evaluated by using as calibrator the Ct values of this gene in the infected, but not treated with Tramesan, samples (control).

### Assay of Tramesan on mammalian cell lines

The analysis were carried out on murine melanoma B16-F10 stabilized cells, grown in DMEM and incubated at 37°C with 5% CO_2_ for different times. An amount of cells/well (1x10^5^) were plated in 12-wells plate and incubated o/n. The day after, the cells were treated with Tramesan 0.38 μM and incubated for 48 h. Subsequently, the cells were centrifuged for 7 min at 1000 rpm and the pellet was washed with PBS. The pellet was then resuspended in 50 μL of 1M NaOH and incubated 1 h at 60°C. The quantity of extracted melanin was determined at 405 nm, with a Perkin-Elmer Lambda 25 UV/Vis spectrometer. Melanin content was calculated by interpolating the results with a standard curve, generated by absorbance of known concentrations of synthetic melanin and corrected for the number of cells. Three determinations were performed in duplicate, the results were expressed as μg of total melanin/number of cells, and values were reported as percentage of control. At the same time, experiments were carried out treating the melanoma cells (about 4 x 10^4^ cells for well) with Tramesan 0.38 μM. The melanoma cells were then incubated at 37°C for 24 and 48 h and the cell counts were performed with light microscopy at these time points. For evaluating gene expression, an amount of B16-F10 stabilized cells (3x10^5^) was plated and incubated. After 24 h of incubation, the cells were treated with Tramesan 0.75 μM and after 6 h from the treatment, the cells were harvested. Before RT-PCR analysis, the cells were washed with PBS and total RNA was isolated using RNeasy Minikit (Qiagen). Subsequently, cDNA was synthesized using oligo-dT primers and ImProm-IITM reverse transcriptase (Promega) according to the manufacturer’s instructions. RT-PCR was carried out in 15 μL (total volume) with SYBR green PCR Master Mix (Bio-Rad) and 200 nM of each primer. The sequences of primers were forward and reverse: β-actin 5’-GACAGGATGCAGAAGGAGATTACT-3’ and 5’-TGATCCACATCTGCTGGAAGGT-3’; nrf-2 for 5’ -CGCTGGAAAAAGAAGTGG- 3’ and rev 5’-AGTGACTGACTGATGGCAGC- 3’. The housekeeping gene β-actin proved as being the most stable after analysis with the Normfinder algorithm (https://moma.dk/normfinder-software). RT-PCR reactions were carried out in triplicate using the Real Time Detection System (iQ5 Bio-Rad) equipped with ICYCLER IQ5 optical system software version 2.0 (Bio-Rad). The condition of thermal cycling were: initial denaturation step at 95°C for 3 min, followed by 40 cycles at 95°C for 10 sec and 60°C for 30 sec.

## Results

### Bio-based purification assays

In a previous study, we assessed that culture filtrates of *T*. *versicolor* inhibited aflatoxin synthesis by enhancing the antioxidant capacity of *A*. *flavus* [33]. Notably, aflatoxin synthesis, as well as other secondary metabolites in pathogenic fungi, is controlled by the cell redox status *via* AP-1 like factors [20,33,34]. Thus, we demonstrated that bioactive compounds present in the culture filtrate of *T*. *versicolor* enhanced Ap1-like gene expression in *A*. *flavus* that, in turn, switched off toxin synthesis [32,35,36].

In order to reduce volumes, and mainly the time needed to verifying aflatoxin inhibition, we set a more handy assay using 96-wells microplates. This is based on the ability of *A*. *flavus* to grow and produce toxins in a small volume, as reported in materials and methods section. Firstly, we separated the main components of a fraction of *T*. *versicolor* culture filtrate particularly active in inhibiting aflatoxins (fraction A), [36]. Fungal secretome is essentially composed of polysaccharides, proteins (glycosylated and not, with enzymatic or different activities), polyphenols and small metabolites [37–40]. In relation to this, considering the fraction A as essentially free from polyphenols, small metabolites and small peptides [36], we separated fractions enriched with polysaccharides or proteins. A scheme of the procedure is present in Figure B in S1 File. Thus, we originated six fraction (from B to G) and we tested them as dry pellets added to fungal medium (1% w/v) with our microtiter-based bioassay (**Table 1**). In this screening, we included also a commercially available “extract” of *T*. *versicolor*, used as diet supplementation (C-TV).

**Table 1:** Inhibition of aflatoxin B1 biosynthesis in *A*. *flavus* 3357 by *T*. *versicolor* culture filtrate fractions. *A*. *flavus* was grown for 3 days into 200-μL multiwells plate, at 30°C in dark conditions and treated with different fractions (B-G) originated from **fraction A** as indicated in the scheme presented into Figure B in S1 File. Commercially available “extract” of *T*. *versicolor*, used as diet supplementation (C-TV) was included too. Results represents the mean of 3 (biological) x 12 (technical) replicates ± SE.

Fractions	Aflatoxin B1 inhibition (%)
Fraction B	76.3 ± 2.2
**Fraction C**	**90.3 ± 3.1**
Fraction D	10.2 ± 0.7
Fraction E	60.2 ± 0.5
Fraction F	58.3 ± 3.5
Fraction G	7.5 ± 3.1
C-TV	5.0 ± 2.2
Fraction A	75.2 ± 1.2

Fraction C proved to be the most efficient in inhibiting aflatoxin synthesis (up to 90%) without a significant fungi static effect (data not shown). Indeed, other fractions had a lower aflatoxin-inhibitory effect whilst preserving a certain degree (up to 50%) of fungal growth inhibition (data not shown). It is possible that for these fractions a certain amount of polysaccharide co-precipitated with proteins that, in turn, could present a slight antimicrobial ability. In relation to this, aflatoxin inhibition may be related also to fungal growth reduction.

The aflatoxin microtiter-based bioassay allowed us to identify fraction C as the one active limiting consistently mycotoxin synthesis by *A*. *flavus*.

### Purification and characterization of fraction C

Fraction C was analysed by ^1^H-NMR and the spectrum showed the typical pattern of saccharidic molecules (data not shown) indicating that its major component was a polysaccharide. A scheme of fraction C fractionation and characterization is reported in Figure C in S1 File In addition, fractions obtained from different *T*. *versicolor* cultivations resulted to contain the same polysaccharide as confirmed by NMR analysis. The polysaccharide produced by *T*. *versicolor* strain C was compared with that present in a *T*. *versicolor* commercial powder by means of composition analysis. The data (Table A in S1 File) indicated that the two samples are very different: Fraction C contained Fuc, Man, Gal and Glc, while the commercial powder contained mainly Man and Glc, a small amount of Gal, but no Fuc. Therefore, these preliminary data suggested that *T*. *versicolor* strain C produced a novel polysaccharide, not present in the commercial sample.

Fraction C was subjected to size exclusion chromatography and three fractions (referred as CI, CII and CIII see Figure D in S1 File) were obtained. They were tested using aflatoxin inhibition in *A*. *flavus* as indicator of biological activity (as described above): fraction CI did not show any effect while fractions CII and CIII presented a significant bioactivity (Figure F in S1 File). These fractions were analysed by ^1^H NMR spectroscopy (Figure G in S1 File) which, a part the 1.3 and 4.2 ppm peaks, due to free lactate often present as contaminant from hands, showed that fractions CII and CIII were structurally identical. Therefore, further 2D NMR experiments were carried out only on fraction CIII, taking advantage of its lower molecular mass. In fact, high-pressure size exclusion chromatography using pullulan standards, (Figure A in S1 File) showed that fraction CII had a molecular mass of about 23000 Da and fraction CIII of about 6000 Da. Compositional analysis of fraction CIII showed Fuc:Man:Gal:Glc in the molar ratios 1.00:2.25:2.22:0.26. Methylation analysis for glycosidic linkages determination showed a rather complex pattern reported in **Table 2**. The presence of 2,6-Gal and 2,6-Hex indicated the occurrence of two branch sites as also confirmed by the presence of terminal non-reducing sugars, t-Fuc and t-Man. The sum of all terminal monosaccharides (t-Fuc + t-Man) is 1.01, in very good agreement with the sum of all branched monosaccharides (2,6-Gal + 2,6-Hex), equal to 0.94. The most important finding is the presence of fucose, an uncommon component of fungal polysaccharides found only in *Mucorales* and in some basidiomycetes [41].

**Table 2:** Glycosidic linkage positions in Tramesan fraction III and in the polysaccharide sample eluted at the void volume (V_0_) of the Bio Gel P2 column after partial hydrolysis.

Monosaccharides	Fraction CIII	Fraction V_0_
t-Fuc	0.12	-
3-Fuc	0.47	0.20
t-Man	0.89	0.91
2-Man	1.00	1.00
6-Gal	0.80	1.76
2,6-Gal	0.66	0.16
2,6-Hex	0.28	0.71

Numbers indicate the position of glycosidic linkages, t-Fuc = terminal non-reducing Fuc, t-Man = terminal non-reducing Man.

**Fig 1:**
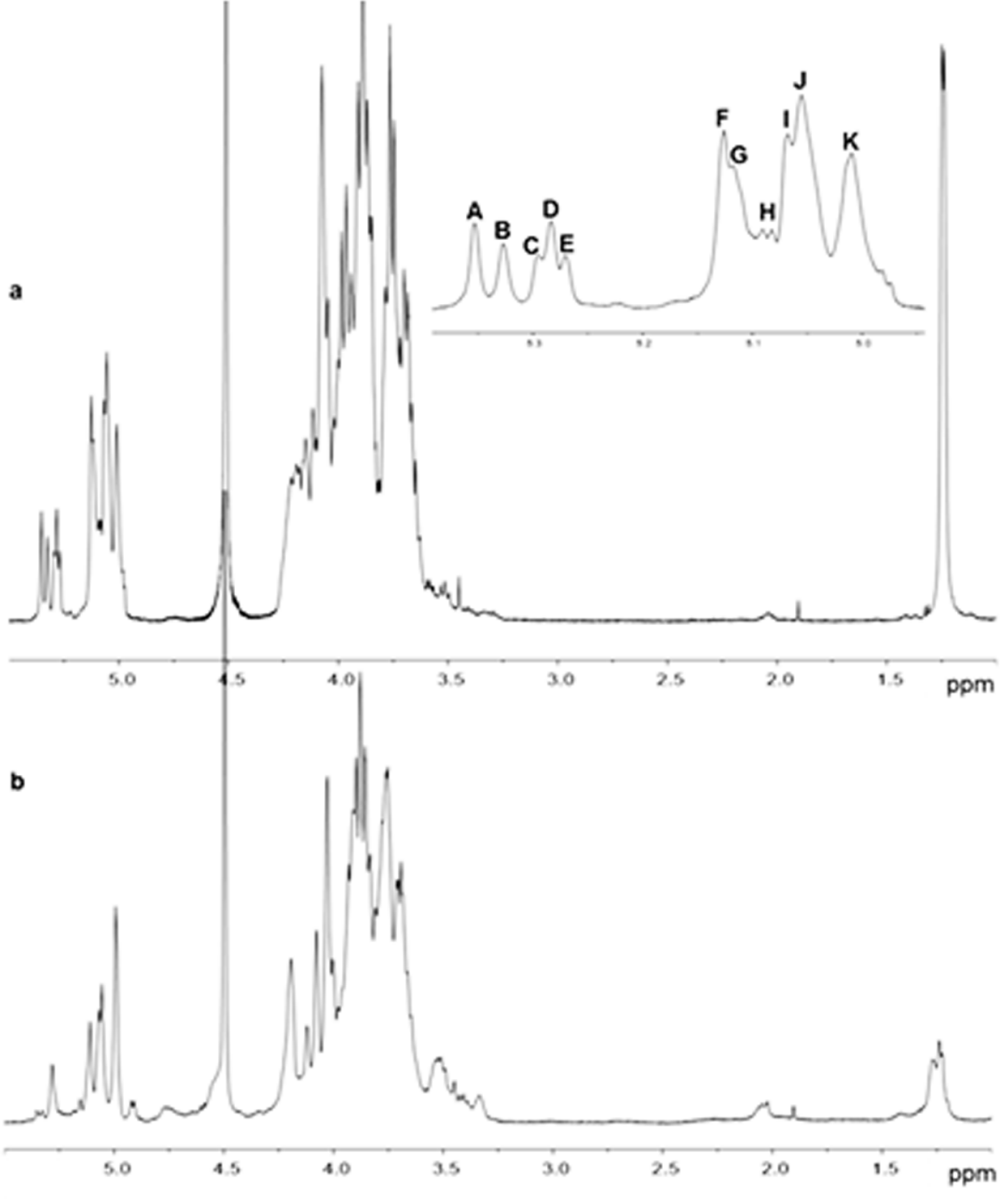
^1^H NMR spectrum (500 MHz, 50°C, D_2_O) of Tramesan. A) Tramesan purified from T. versicolor culture broth (fraction CIII). The enlargement of the anomeric protons resonance ppm range is shown as insert where the most intense anomeric protons are named from A to K. B) ^1^H NMR spectrum (500 MHz, 50°C, D_2_O) of the total excluded high molecular mass fraction (V_0_) obtained by partial hydrolysis of Tramesan.

Since the ^1^H NMR spectrum indicated the presence of O-acetyl groups (Figure F in S1 File) which increase the complexity of the spectrum [42], fraction CIII was de-O-acetylated for further NMR analysis. The ^1^H NMR spectrum (**Fig 1A**) is typical of a polysaccharide: the anomeric proton region, between 5.5–4.8 ppm, (**inset of Fig 1A**) exhibited a complex pattern, reflecting the glycosidic linkage data and suggesting the absence of regular repeating units. The resonance at 1.25 ppm was attributed to the methyl groups of 6-deoxy residues, confirming the presence of Fuc.

After inspection of the ^1^H NMR spectrum and of the COSY plot, the most intense anomeric protons were named from A to K (inset of Fig 1A) in order of decreasing chemical shift, and TOCSY, NOESY and HSQC experiments were recorded. Heavy overlapping of most peaks prevented complete chemical shifts assignments; nevertheless, some useful information could be gained.

Resonances belonging to H1 and H2 of each spin system were easily identified from the COSY plot. Taking advantage of the small *J*_H1-H2_ coupling constant of Man, which results in the absence of magnetisation transfer from H2 to H3 in the TOCSY plot, this monosaccharide was easily identified with signals **A**-**E**, **F**, and **J.** Resonances of carbon nuclei linked to the respective protons were then assigned from the HSQC plot (Figure G in S1 File) and the data collected can be found in Table B in S1 File. Resonances from **A** to **E** had H2s at relatively low field and C2s chemical shifts were shielded with respect to values for unbound Man [43–45], indicating engagement in glycosidic linkage; therefore, they were attributed to 2-Man residues. The very small difference of chemical shifts of these Man residues suggested that they may be part of oligosaccharide side chains of different sizes [46], thus experiencing slightly different electronic environments. The signals **F** and **J** were tentatively assigned to t-Man. Spin system **G** was completely assigned and attributed to 3-Fuc, following the magnetisation transfer in the TOCSY plot starting from H1 as well as from H6, which is well separated from the other signals. Furthermore, C6 nuclei engaged in glycosidic linkages were clearly detected in the HSQC spectrum as negative peaks (Figure G in S1 File**, blue traces**), shifted to higher ppm values with respect to the corresponding unbound residue (from about 62 to about 67.5 ppm), confirming the methylation analysis findings.

**Fig 2:**
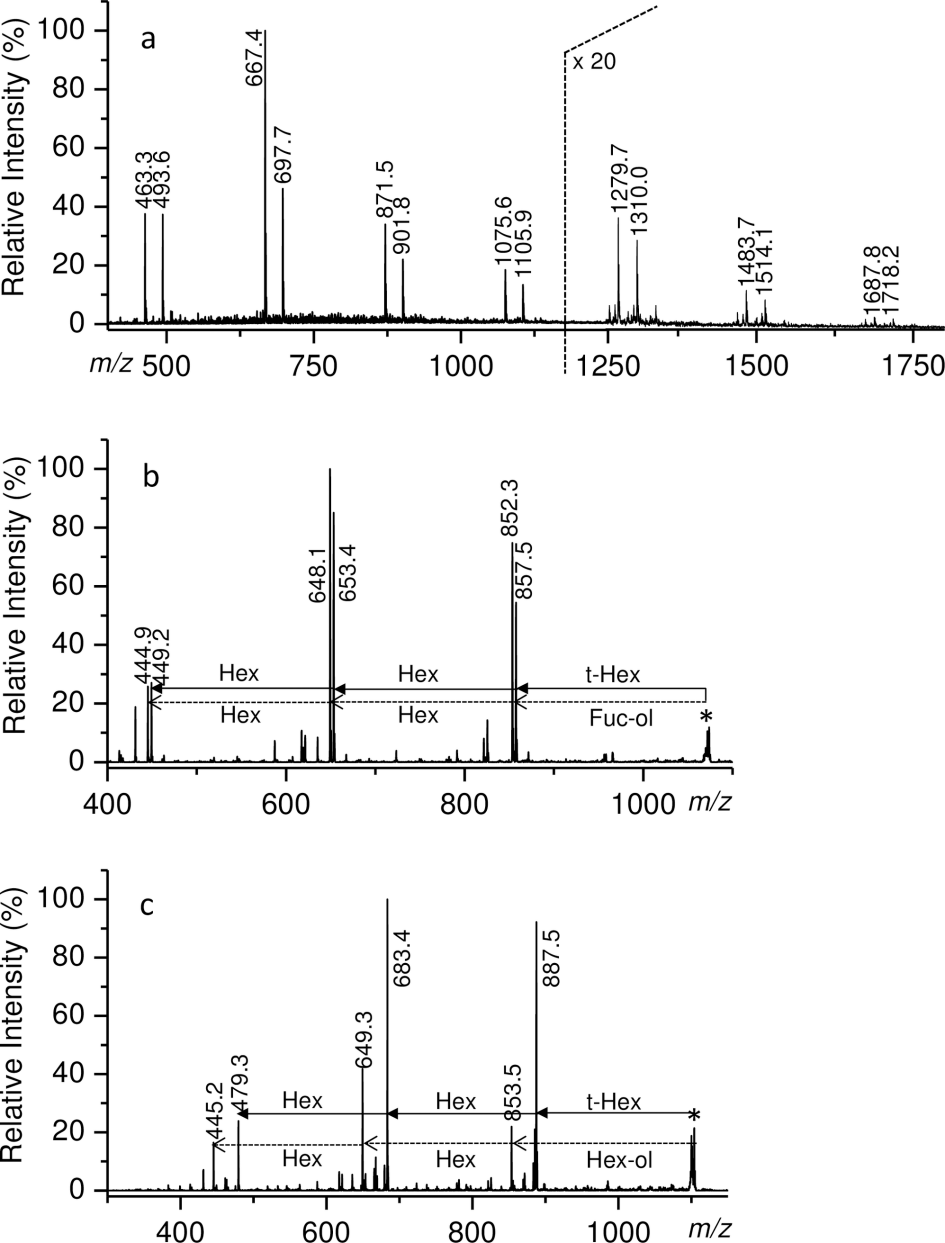
**A) ESI-MS spectrum of the reduced and permethylated oligosaccharide mixture obtained by partial hydrolysis, ESI-MS**^**2**^ **spectra of the parent ions at 1075.6 (B) and 1105.9 (C) *m/z*.** The fragmentation schemes (B and C) starting from the non-reducing end are reported with full lines, those starting from the reducing end with dotted lines. Fuc-ol and Hex-ol stands for fucitol and hexitol, respectively.

**Fig 3:**
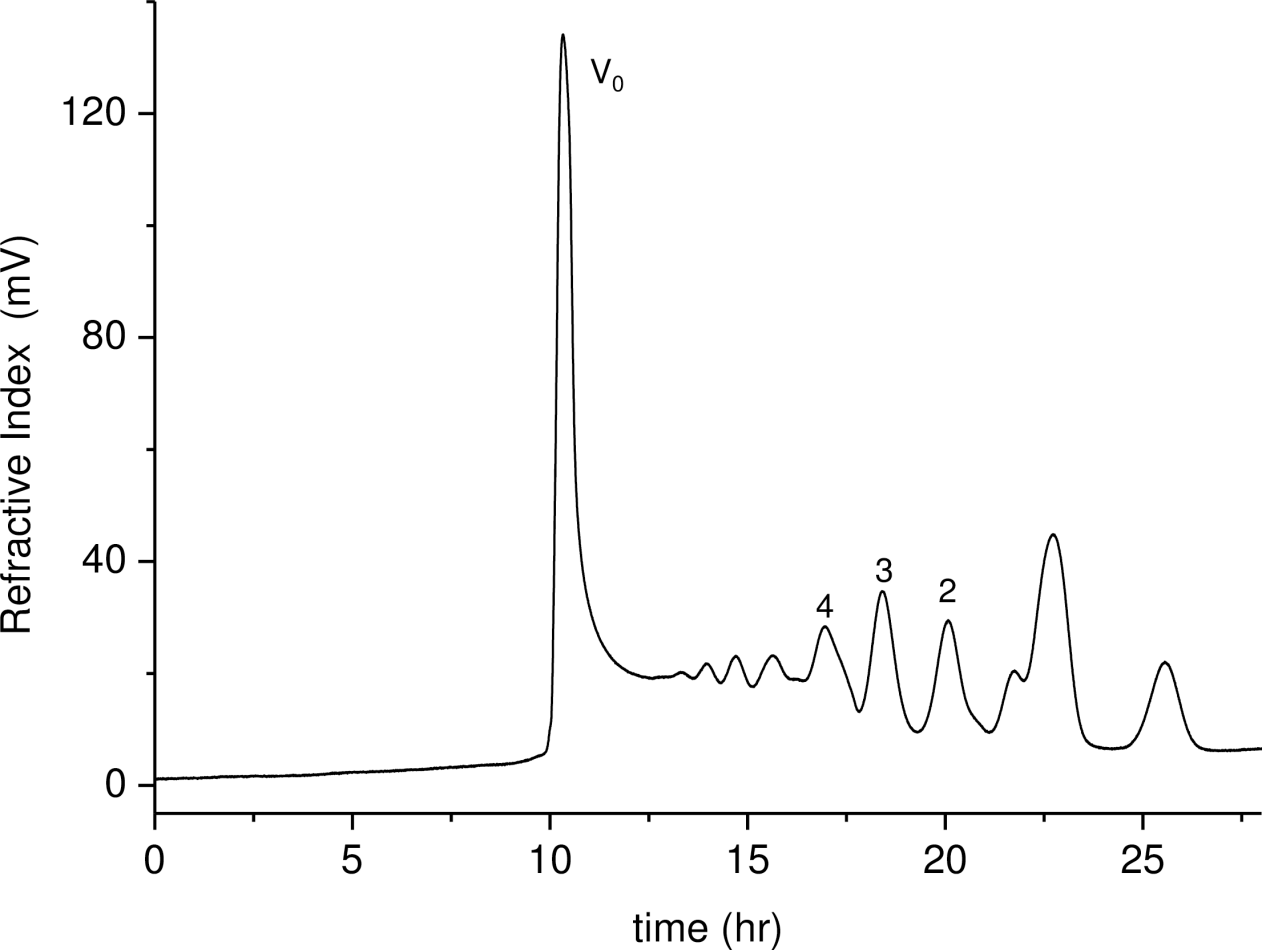
Size exclusion chromatographic pattern of the mixture obtained from Tramesan by partial hydrolysis. V_0_ indicates the total excluded high molecular mass fraction. Numbers 2–4 refer to sugar residues in oligosaccharides separated by the Bio Gel P2 resin.

In order to gain more insights on the primary structure of Tramesan, a sample was subjected to partial hydrolysis, taking advantage of the more labile glycosidic linkage of Fuc residues. A small amount of hydrolysate was subjected to ESI-MS and the spectrum (data not shown) indicated a series of oligosaccharides from disaccharides to octasaccharides. The sample mixture was then reduced with sodium borohydride and permethylated. ESI-MS (**Fig 2**A) showed pairs of (M + Na)^+^ parent ions corresponding to oligosaccharides, differing for the presence of one Fuc residue in place of one Hex residue. MS^2^ (**Fig 2B and 2C**) of the pentasaccharides gave a good fragmentation pattern establishing the exclusive reducing terminal position of the Fuc residues. The hydrolysate was separated by size exclusion chromatography on a Bio Gel P2 column (**Fig 3**); fractions belonging to same peak were pooled together and subjected to NMR spectroscopy.

**Fig 4:**
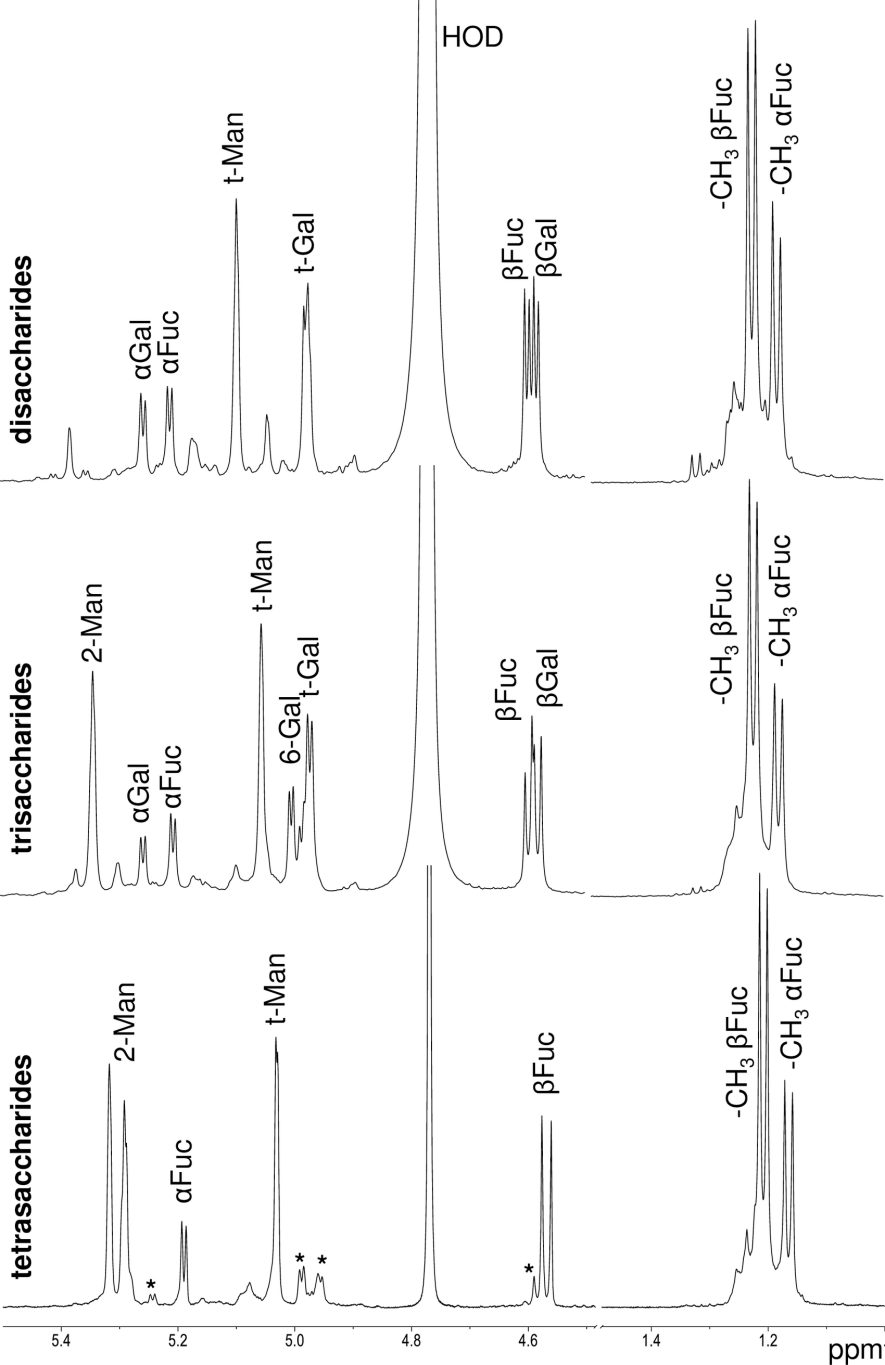
^1^H NMR spectra (500 MHz, 50°C, D_2_O) of oligosaccharides obtained after partial hydrolysis and SEC-chromatography. From top to bottom: A) disaccharides, B) trisaccharides and C) tetrasaccharides.

The expansions of the anomeric and methyl regions of the ^1^H NMR spectrum of the disaccharides mixture is reported in **Fig 4A**; only the major signals were considered and they were assigned after examining the COSY, TOCSY, NOESY and HSQC spectra as well. The methyl doublets at 1.23 and 1.18 ppm belong to the H6 of Fuc residue and reflect the anomeric equilibrium between α- and β-forms, thus confirming its reducing end position.

The resonances at 5.21 and 4.60 ppm were assigned to the α- and β- anomeric forms of Fuc, while the resonances at 5.26 and 4.59 ppm were attributed to the α- and β- anomeric forms of Gal, thus showing two different reducing ends, and therefore, two different disaccharides. Based on the *J*_H1-H2_ coupling constants, the signals at 5.09 and 4.97 ppm were assigned to Man and Gal, respectively. The coupling constant *J*_H1-H2_ = 3.5 Hz for the Gal signal at 4.97 ppm established its α anomeric configuration, while the determination of the Man configuration could not be achieved, because of the very small and similar *J*_H1-H2_ of α- and β- Man. Integration values of the peak areas are reported in **Table 3** and they are consistent with two different disaccharides in equimolar amounts.

**Table 3:** Chemical shifts assignment and area integration (Int) values for the anomeric protons and methyl protons of disaccharides, trisaccharides and tetrasaccharides obtained from partial hydrolysis of Tramesan.

DISACCHARIDES		TRISACCHARIDES		TETRASACCHARIDES	
Assignment (ppm)	Int values	Assignment (ppm)	Int values	Assignment (ppm)	Int values
		2-Man (5.34)	1.00	2-Man (5.32)	1.00
				2-Man (5.29)	1.11
α-Gal (5.26)	0.38	α-Gal (5.26)	0.31		
α-Fuc (5.21)	0.43	α-Fuc (5.20)	0.45	α-Fuc (5.19)	0.41
t-Man (5.10)	1.00	t-Man (5.05)	1.23	t-Man (5.03)	1.14
t-Gal (4.98)	1.03	6-Gal (5.00)	1.89		
		6-Gal (4.99)			
		t-Gal (4.97)			
β-Fuc (4.60)	0.68	β-Fuc (4.60)	1.38	β-Fuc (4.57)	0.76
β-Gal (4.59)	0.72	β-Gal (4.58)			
-CH_3_ of Fuc (1.23, 1.18)	4.00	-CH_3_ of Fuc (1.22, 1.18)	3.77	-CH_3_ of Fuc (1.21, 1.16)	4.23

Inspection of the COSY, TOCSY and HSQC spectra led to the assignment of most of the peaks (see Table C in S1 File); in the HSQC plot (data not shown) C6 signals engaged in glycosidic linkages were visible at about 67 ppm, in agreement with 6-linked Gal. NOESY spectrum (data not shown) showed a strong correlation between H1 of Man and H3 of Fuc, thus establishing the structure Man-(1→3)-Fuc. Therefore, the second disaccharide is α-Gal-(1→6)-Gal. These experimental data were corroborated by the output of the CASPER computer program for the two disaccharides sequences [47,48].

The expansions of the anomeric and methyl regions of the ^1^H NMR spectrum of the trisaccharides mixture is reported in **Fig 4B**. By comparison with the ^1^H NMR spectrum of the disaccharides and resorting to chemical shifts values from the literature, anomeric protons were easily assigned, as shown in **Fig 4C**, and these assignments were also corroborated by COSY, TOCSY, HSQC and NOESY spectra (data not shown). Integration values of the main peaks are reported in **Table 3** and the data collected suggested the presence of two main trisaccharides in roughly equimolar amounts: Man-(1→2)-Man-(1→3)-Fuc and Gal-(1→6)-Gal-(1→6)-Gal. Therefore, the obtained trisaccharides are consistent with the structure of the disaccharides with the addition of one further unit: Man and Gal, respectively.

The enlargement of the anomeric and methyl regions of the ^1^H NMR spectrum of the tetrasaccharides fraction showed two sets of signals having very different intensities (**Fig 4C**); the most intense resonances were assigned by comparison with both literature values and with the di- and trisaccharides spectra. Integration data are reported in **Table 3**. The data collected indicated that in the tetrasaccharide peak the main oligosaccharide present has the structure Man-(1→2)-Man-(1→2)-Man-(1→3)-Fuc, while signals attributable to Gal residues (marked with a star in Fig 3C), and, by analogy with di- and tri-saccharides structures, likely belonging to a galactotetraose, were much less intense.

The fraction eluted at the void volume (V_0_) of the Bio Gel P2 column obtained from the partial hydrolysis reaction, containing high molecular mass oligomers, was subjected to linkage analysis and ^1^H NMR spectroscopy. The results of linkage analysis are reported in **Table 2**: t-Fuc was not detected, while the intensity of 3-Fuc dramatically decreased, in agreement with its presence as reducing end residue in the oligosaccharides. The total amount of branched residues (2,6-Gal + 2,6-Hex = 0.87) is in very good agreement with the amount of t-Man (0.89). The residue 2,6-Gal also decreased with the concomitant increase of 6-Gal, thus indicating that partial hydrolysis removed side chains attached on C2 of Gal, producing the oligosaccharides described above. Therefore, the main chain of Tramesan is a 1–6 linked galactan with (Man)_n_-Fuc branches in position 2, and according with glycosidic linkage analysis (Table 2, fraction CIII), some side chains can consist only of t-Fuc. Linkage analysis showed that t-Man and 2-Man were present in the V_0_ fraction, indicating that they are not confined in the 2-linked Gal branches; together with the relative increase of 2,6-Hex (Table 4, fraction V_0_), data might indicate the presence of (Hex)_n_-(Man)_m_ branched sequences, either composing a second polysaccharide, or bound to the above described galactomannan. Furthermore, the gas-chromatographic retention time of 2,6-Hex peak was identical to that of a 2,6-Man standard from our laboratory (Figure H in S1 File), and the absence in fraction V_0_^1^H-NMR spectrum (**Fig 1B)** of resonances typical of Glc units [43] strongly suggested to identify 2,6-Hex with 2,6-Man.

**Table 4:** Biological activity of Tramesan on *A*. *flavus* and *A*. *parasiticus*. *In vitro* culture of *A*. *flavus* and *A*. *parasitucus* under aflatoxin permissive conditions (PDB, 30°C) were treated or not (control) with 0.38 μM Tramesan and incubated for 7 days. Aflatoxin B1 production, evaluated by LC-MS/MS, and fungal growth, evaluated by weighting dried mycelia, in Tramesan-treated cultures, were normalised for non-treated ones and the percentage of inhibition calculated consequently. Mycelia was used to evaluate the expression, calculated by 2^-ΔΔCt^ method in RT-PCR, of the oxidative stress related transcription factors Af*yapA* and Ap*yapA* and the superoxide dismutase encoding gene *sod1* in *A*. *flavus* and *A*. *parasiticus*, respectively.

Fungal species	treatment	Aflatoxin B1 (ppb)	% of Aflatoxin B1 inhibition compared to untreated control	Fungal growth (mg/mL d.w.)	Ap-1 *like*	*sod1*
*A*. *flavus*	*control*	125.2 ± 2.5	95.2	5.1 ± 0.6	2.1 ± 0.3	25.2 ± 3.2
	*Tramesan 0*.*38 µM*	6.02 ± 0.2		5.2 ± 0.5		
*A*. *parasiticus*	*control*	185.5 ± 7.2	98.7	4.5 ± 0.2	2.5 ± 0.2	22.3 ± 4.1
	*Tramesan 0*.*38 µM*	2.4 ± 0.5		4.4 ± 0.8		

The ^1^H NMR spectrum of fraction V_0_ in **Fig 1B** showed interesting changes. The methyl groups of Fuc greatly decreased in intensity, in agreement with methylation analysis. In the anomeric region, H1 resonances of **A**, **B**, **C**, and **E** of Man residues substantially decreased, in agreement with the removal of Man-containing oligosaccharides, linked on position 2 of Gal residues. Signals **F** (t-Man) and **G** (3-Fuc) greatly decreased in intensity, while resonance **H** disappeared, in agreement with its tentative assignment to t-Fuc. Other changes occurred in the region 5.2–4.8 ppm, but no precise information could be obtained on the type of monosaccharides involved.

**Fig 5:**
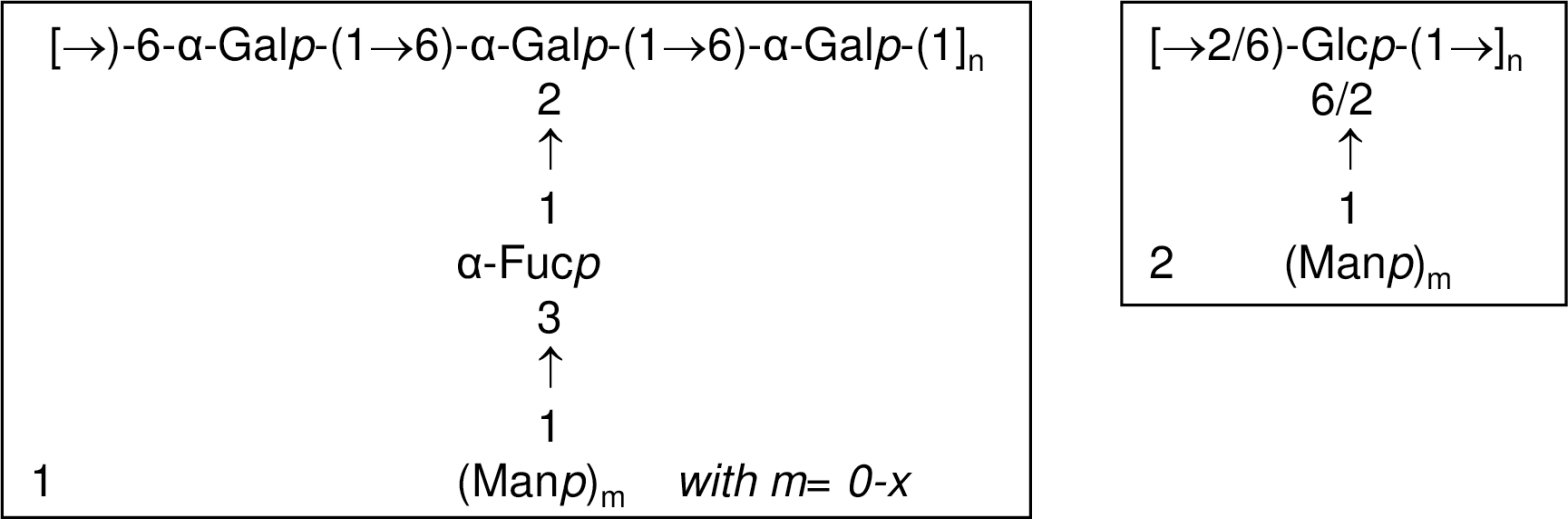
Proposed structure for Tramesan. Scheme 1 and 2 can be part of the same polysaccharide or forming repeating units of different polymers.

Although for a detailed structural definition Tramesan needs to be deeper investigated, the data obtained in this study on the biologically active polysaccharidic fractions suggested that it consists of two sequences that may or may be not part of a single polymer, similar to other fungal polysaccharides [49]. The proposed structures are shown in **Fig 5**.

### Biological effects

Purified Tramesan was firstly tested on *A*. *flavus* and *A*. *parasiticus* for its inhibiting effect on fungal growth and aflatoxin B1 production. Tramesan was used at a concentration 0.38 μM as suggested by Scarpari *et al*. [36]; the amount utilised, amended from fungal culture, did not significantly affected the fungal growth of both strains whereas, the aflatoxin B1 production was markedly inhibited (p<0.001, **Table 4**). Considering that aflatoxin biosynthesis is under the control of oxidative stress [34,50], the expression of Af*yapA* and Ap*yapA*, oxidative stress related transcription factors that control the synthesis of aflatoxin upstream to the aflatoxin regulator AflR [20,34] and of the superoxide dismutase encoding *sod1* in *A*. *flavus* and *A*. *parasiticus*, respectively, was monitored. It was observed that Tramesan significantly triggered the expression of Af*yapA* and of *sod1* (**Table 4**).

To check if Tramesan could elicit antioxidant response in other biological systems, thus acting as a *pro* antioxidant molecule, we tested its effect in hampering the necrotrophic progression of a foliar pathogen of durum wheat, *Parastagonospora nodorum*. This pathogen produces a necrotrophic effector that also act by increasing ROS production at the interface for facilitating tissue degeneration and cell death [51]. Thus, we pre-treated durum wheat leaves *in greenhouse* with a suspension of Tramesan 48 h before inoculating the pathogen. Concomitantly, we tested the antifungal activity of Tramesan on pure *P*. *nodorum* culture. Wheat plants treated with Tramesan resulted more protected from *P*. *nodorum* infection compared to untreated ones; interestingly, Tramesan had no appreciable effect on fungal growth under *in vitro* conditions (data not shown), whereas *in planta* limited its growth. We here suggest that Tramesan enhanced some ROS-scavenging ability (e.g. *PR9*) of wheat plant leaves (**Table 5**) disabling necrotrophic weapons for triggering PCD and causing disease.

**Table 5:** Effect of Tramesan on wheat leaves infected with *P*. *nodorum*. Durum wheat leaves of an Italian commercial variety were sprayed with a solution of 0.38μM Tramesan and after 48 h, inoculated with 10^5^ conidia of *P*. *nodorum*; other plants were inoculated with the pathogen but not pre-treated with Tramesan (Infected control). Disease severity was quantified by using Liu’ scale (necrotic spot extension) whereas relative expression of the peroxidase-encoding, PR-9 gene, was calculated using the 2^-ΔΔCt^ method. Fungal growth was assessed by qPCR using *P*. *nodorum* specie-specific primers.

Durum wheat leaves	Necrotic spot	Fungal growth	*PR-9 (peroxidase)*
	rated by Liu’ scale^1^	qPCR (ng fungal DNA/μg total DNA)	mRNA relative expression (2^-ΔΔCt^)
Infected control	4	80.1 ± 2.2	1
Tramesan	0	5.0 ± 0.5	64.2 ± 2.1

^**1**^*Liu et al., 2004 [30]*

To validate the widespread ability of Tramesan to behave as a *pro* antioxidant molecule, we also tested its effect on a murine cell line of melanoma (B16). In this case, the expected effect is a limitation in cell growth since cancer cells express high level of intrinsic oxidative stress that normally boosts their division [52]. Notably, we aimed at checking if Tramesan was able to enhance the ROS scavenging ability of these cutaneous murine cells. In relation to this, we quantified the amount of an antioxidant molecule (melanin) and tested the expression of *Nrf-2* mRNA, since *Nrf-2* protects melanocytes against the harmful ROS effects [53]. Cells treatment with 0.38 μM Tramesan increased melanin content of about two-folds whilst enhancing *Nrf-2* expression and consistently reducing cell growth (**Table 6**).

**Table 6:** Effect of Tramesan on murine cell lines of melanoma (B16). Murine cell lines of melanoma (B16) were treated with 0.38 μM Tramesan or untreated (control). The amount of melanin was calculated by spectrophotometer (Abs_405_), normalised for untreated samples and the percentage of its increase was evaluated and compared to untreated samples. Cell growth was calculated by counting the number of viable cells. The results are the mean ± SD of 5 separate experiments Relative expression of the *Nrf-2* gene normalised on untreated cells was calculated using the 2^-ΔΔCt^ method.

	Melanin	Cell growth	*Nrf-2*
	Percentage of increase compared to untreated samples (%)	Percentage of reduction of cell number (%)	mRNA relative expression (2^-ΔΔCt^)
*Tramesan*	97.5 ± 4.3	83.7 ± 2.2	9.5 ± 0.2

## Discussion

Fungal glycans may act as eliciting molecules in host cells. In addition, some fungi produce and secrete glycans to be recognised [54]. Recently, some oligomers of chitin were found to be actively released in the interface between fungal symbionts and their host to “facilitate” recognition processes and repress host defence [21,55]. Apart lignin degrading enzymology, few reports actually concern the biology of *T*. *versicolor*. In a previous study, the semi-purified culture filtrate of *T*. *versicolor* containing molecules with a supposed molecular mass (MM) >3.0 kDa showed a particular ability in enhancing antioxidant activity in *A*. *flavus*, dramatically inhibiting the biosynthesis of aflatoxins [36]. In relation to this, we exploited the ability of the semi-purified culture filtrate of *T*. *versicolor* to inhibit aflatoxin biosynthesis as a fast and reliable method to detect active principles present into the MM>3.0 kDa fraction (fraction A in this study). We found that this mushroom produces an exo-polysaccharide that is not simply “leaked” by the complex fungal cell wall, but is secreted into the environment with a still unknown biosynthetic and transportation pathways and, overall, with an unknown biological function. In the present study, we provide some hints for elucidating the latter. Tramesan is a branched fucose-enriched fungal polysaccharide of about 23 kDa with a probable “repetitive” scheme of monosaccharide sequence in the linear (α-1,6-Gal)_n_ backbone as well as in the lateral chain Man-(1→2)-Man-(1→3)-Fuc. It could be suggested that host cells may recognize fungi from their saccharidic “barcode” composed by a scheme of repetitive units in which the single unit acts–*de facto–*as signalling molecule. This was recently confirmed for signal factors in fungal mycorrhizae that are composing lipochitooligosaccharides [56] and chitooligosaccharides [54]. These fungal complex glucans may elicit calcium waves in different systems and Tramesan, when administered to murine melanoma cell culture, displays this effect too (data from our laboratories). Thus, what we suggest here is that Tramesan may act for *T*. *versicolor* as an “informative” molecule able to modify the relation with other organisms into its trophic niche.

In our study, we demonstrated that Tramesan might elicit similar responses in different organisms. Notably, in previous reports non-purified Tramesan proved able to induce the activation of antioxidant defences into mycotoxin producing fungi [33,36]. Several mycotoxigenic fungi are sensitive to variation in the cell redox balance and many exploit augmentation in circulating ROS by vehiculating this oxidative power into toxin synthesis [50,57]. Thus, the hypothesis ongoing after these results proposes Tramesan as a *pro* antioxidant molecule, able to trigger the expression of oxidative stress related transcription factors (e.g. *Nrf-2* like as well as *yAP-1* like factors) and, in turn, re-establish the proper cell redox balance and “subtracting” oxidative power that boosts toxin synthesis. Since ROS are often the molecular trigger for opening different responses such as defence and differentiation in plant and animals [58–60], we tested two different “models”: the defence reaction of a plant to a necrotrophic pathogen (*P*. *nodorum* x *T*. *turgidum* subsp. *durum*) and the melanin differentiation into murine melanoma. As stated previously, these mechanisms are triggered, or at least accompanied, by a cell redox unbalance due to oxidative burst [53,60]. Tramesan enhances the defence ability of durum wheat as well as melanin biosynthesis in melanoma. Notably, this polysaccharide induces the expression of oxidative stress defence-related genes such as peroxidases and *Nrf-2*. Even in these organisms as well as in mycotoxin producing fungi, Tramesan can elicit an antioxidant response, probably, by manipulating gene expression. We here suggest that, Tramesan could be recognized by specific receptors that, in turn, activate pathways leading to an antioxidant response. As it is known, several receptors in different organisms act as ligands of fungal glycans and, in turn, trigger various responses ranging from innate immunity to cell death or symbiosis [5,12,61]. Tramesan could act as ligand for a still unknown inter-kingdom conserved receptor able to control antioxidant responses.

In conclusion, we present for the first time the partial elucidation of the structure of Tramesan, a branched fungal glycan secreted into the environment by the lignin degrading fungus *T*. *versicolor*. This compound is able to act as a *pro* antioxidant in different organisms. By enhancing the “natural” antioxidant defences of the “hosts”, Tramesan could represent a useful tool for different challenges in different “contexts” in which limiting ROS production represents a solution for defusing harmful situations such as toxins release into foodstuff, necrotic lesions into plant crops or growth of cancer cells.

## Supporting information

S1 File **Figure A.** HP-SEC calibration plot obtained with nine pullulan standards with molecular masses in the range 1.7x10^6^–5.9x10^3^, used to evaluate Tramesan fraction molecular masses.
**Figure B.** Scheme summarizing the purification steps applied for the obtainment of the different fractions (B-G) used in the aflatoxin inhibition bioassays.
**Figure C.** Scheme of the characterization of the bioactive polysaccharide fraction C.
**Figure D.** Size exclusion chromatography on a Sephacryl S-300 column of the filtrate C from *T*. *versicolor* TV117 cultures. The three obtained fractions (CI, CII, CIII) are indicated.
**Figure E.** Aflatoxin inhibition by the different SEC-separated fractions (I-III).
**Figure F.** ^1^H-NMR spectra of the fractions II (red) e III (blue) obtained after size exclusion chromatography on a Sephacryl S-300 column.
**Figure G.** HSQC plots of Trametan fraction III solution recorded at 50°C. A) Expansion of the anomeric region and B) expansion of the ring region. Assignments as reported in **S2 Table**, relative to acetone (2.225 ppm for ^1^H and 31.07 ppm for ^13^C). H6, C6 of hexoses are in blue: those belonging to 6-linked hexoses resonate at about 67 ppm, those that are not linked at about 62 ppm.
**Figure H.** Comparison of GLC elution profiles between PMAA derivatives of Tramesan and a sample containing 2,6-Man. The peaks of interest are marked with asterisks.
**Table A.** Composition analysis of the fraction C containing the polysaccharide produced by *T*. *versicolor* Tv117 (PLS 117) and of a *Trametes versicolor* commercial powder sample (CP). Results are expressed in weight %.
**Table B.** ^1^H and ^13^C chemical shift assignments of Tramesan, referred to acetone (2.225 ppm for ^1^H and 31.07 ppm for ^13^C).
**Table C.** ^1^H and ^13^C chemical shift assignments of the disaccharides obtained from partial hydrolysis of Tramesan. Signals are referred to acetone (2.225 ppm for ^1^H and 31.07 ppm for ^13^C).
(DOCX)
