# Supplementary material for: Tramesan, a novel polysaccharide from Trametes versicolor. Structural characterization and biological effects
Source: PLoS One. 2017 Aug 22;12(8):e0171412. doi: 10.1371/journal.pone.0171412 (PMC5567496; doi:10.1371/journal.pone.0171412)
Supplement: S1 File — Figure A. HP-SEC calibration plot obtained with nine pullulan standards with molecular masses in the range 1.7x10^6^–5.9x10^3^, used to evaluate Tramesan fraction molecular masses. Figure B. Scheme summarizing the purification steps applied for the obtainment of the different fractions (B-G) used in the aflatoxin inhibition bioassays. Figure C. Scheme of the characterization of the bioactive polysaccharide fraction C. Figure D. Size exclusion chromatography on a Sephacryl S-300 column of the filtrate C from T. versicolor TV117 cultures. The three obtained fractions (CI, CII, CIII) are indicated. Figure E. Aflatoxin inhibition by the different SEC-separated fractions (I-III). Figure F. 1H-NMR spectra of the fractions II (red) e III (blue) obtained after size exclusion chromatography on a Sephacryl S-300 column. Figure G. HSQC plots of Trametan fraction III solution recorded at 50°C. A) Expansion of the anomeric region and B) expansion of the ring region. Assignments as reported in S2 Table, relative to acetone (2.225 ppm for 1H and 31.07 ppm for 13C). H6, C6 of hexoses are in blue: those belonging to 6-linked hexoses resonate at about 67 ppm, those that are not linked at about 62 ppm. Figure H. Comparison of GLC elution profiles between PMAA derivatives of Tramesan and a sample containing 2,6-Man. The peaks of interest are marked with asterisks. Table A. Composition analysis of the fraction C containing the polysaccharide produced by T. versicolor Tv117 (PLS 117) and of a Trametes versicolor commercial powder sample (CP). Results are expressed in weight %. Table B. 1H and 13C chemical shift assignments of Tramesan, referred to acetone (2.225 ppm for 1H and 31.07 ppm for 13C). Table C. 1H and 13C chemical shift assignments of the disaccharides obtained from partial hydrolysis of Tramesan. Signals are referred to acetone (2.225 ppm for 1H and 31.07 ppm for 13C). (DOCX) [file pone.0171412.s001.docx]

**Tramesan, a Novel Polysaccharide from *Trametes versicolor*.**

**Structural Characterization and Biological Effects.**

Marzia Scarpari, Massimo Reverberi, Alessia Parroni, Valeria Scala, Corrado Fanelli, Chiara Pietricola, Slaven Zjalic, Vittoria Maresca, Agostino Tafuri, Maria R. Ricciardi, Roberto Licchetta, Simone Mirabili, Aris Sveronis, Paola Cescutti, Roberto Rizzo

| Pullulan molecular mass  (kDa) |
| --- |
| 1660 |
| 788 |
| 404 |
| 212 |
| 112 |
| 47.3 |
| 22.8 |
| 11.8 |

**Figure A.** HP-SEC calibration plot obtained with nine pullulan standards with molecular masses in the range 1.7x10^6^ – 5.9x10^3^, used to evaluate Tramesan fraction molecular masses.

**
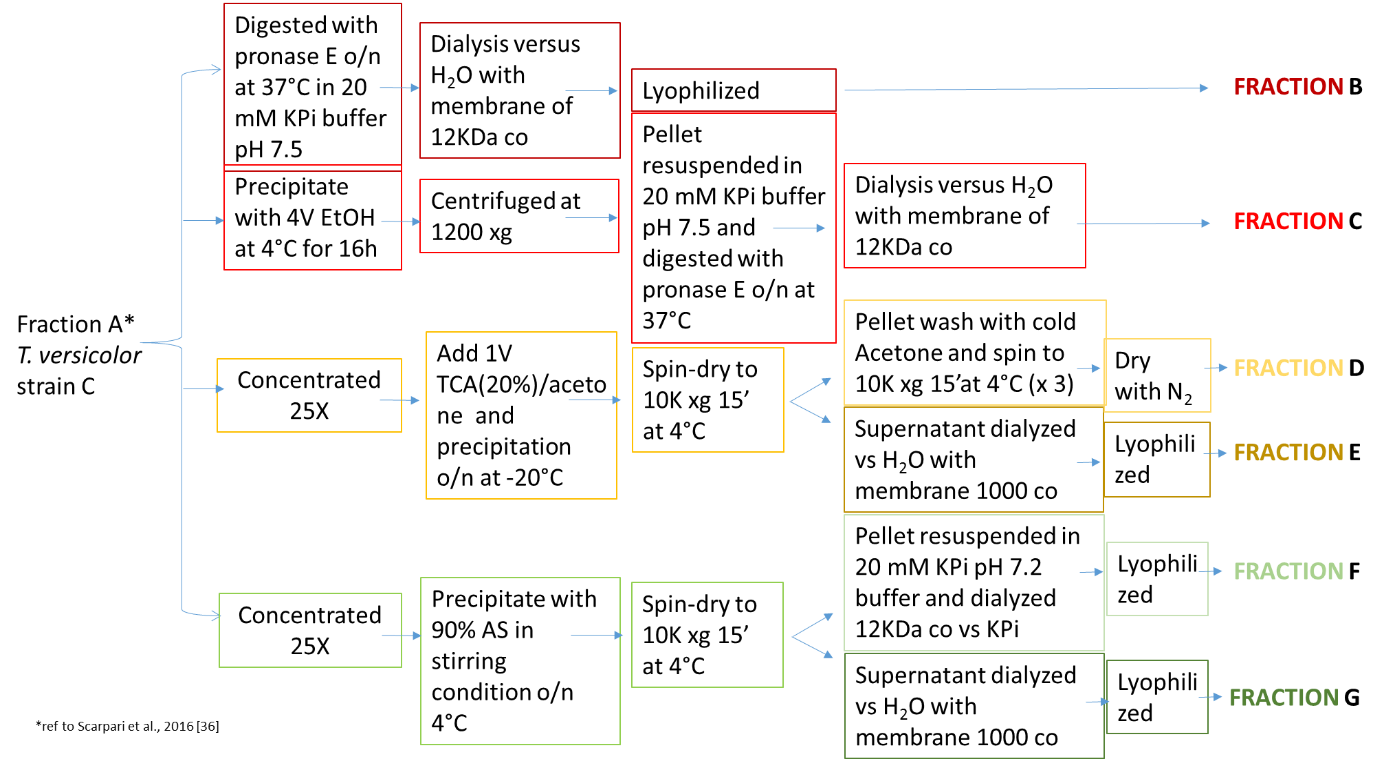
**

**Figure B.** Scheme summarizing the purification steps applied for the obtainment of the different fractions (B-G) used in the aflatoxin inhibition bioassays.

**Table A.** Composition analysis of the fraction C containing the polysaccharide produced by *T. versicolor* Tv117 (PLS 117) and of a *Trametes versicolor* commercial powder sample (CP). Results are expressed in weight %.

| monosaccharide | Sample | |
| --- | --- | --- |
|  | PLS TV117 | CP |
| Fuc | 1.13 | - |
| Man | 19.72 | 48.10 |
| Gal | 17.18 | 1.41 |
| Glc | 61.98 | 50.49 |


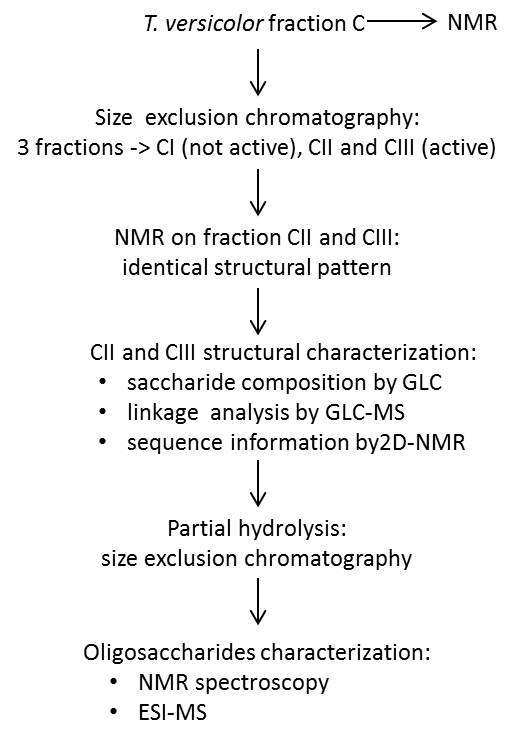


**Figure C.** Scheme of the characterization of the bioactive polysaccharide fraction C.

**Figure D.** Size exclusion chromatography on a Sephacryl S-300 column of the filtrate C from *T. versicolor* TV117 cultures. The three obtained fractions (CI, CII, CIII) are indicated.


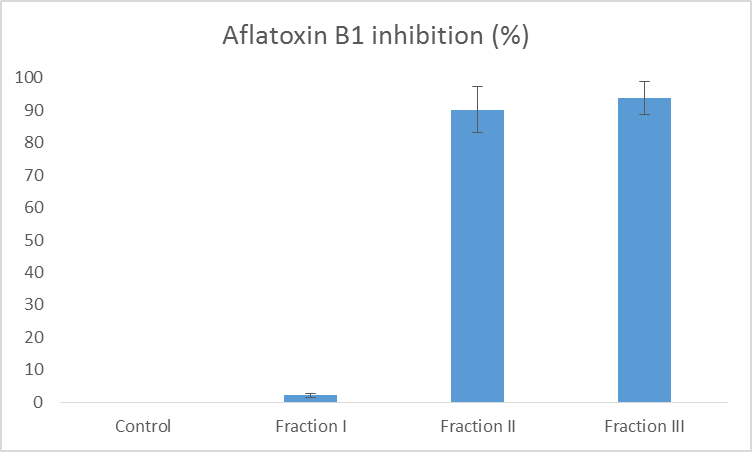


**Figure E.** Aflatoxin inhibition by the different SEC-separated fractions (I-III)

ppm

**Figure F.** ^1^H-NMR spectra of the fractions II (red) e III (blue) obtained after size exclusion chromatography on a Sephacryl S-300 column.


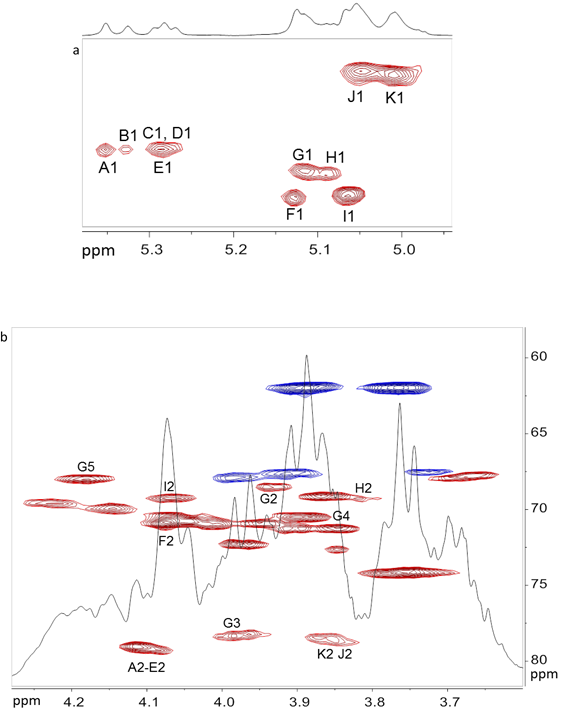


**Figure G.** HSQC plots of Trametan fraction III solution recorded at 50 °C. A) Expansion of the anomeric region and B) expansion of the ring region. Assignments as reported in **S2 Table**, relative to acetone (2.225 ppm for ^1^H and 31.07 ppm for ^13^C). H6, C6 of hexoses are in blue: those belonging to 6-linked hexoses resonate at about 67 ppm, those that are not linked at about 62 ppm.

**Table B.** ^1^H and ^13^C chemical shift assignments of Tramesan, referred to acetone (2.225 ppm for ^1^H and 31.07 ppm for ^13^C).

| **Residues** | **Nucleus** | **Chemical shifts (ppm)** | | | | | |
| --- | --- | --- | --- | --- | --- | --- | --- |
|  |  | **1** | **2** | **3** | **4** | **5** | **6** |
| **A** | ^1^H | 5.35 | 4.12 |  |  |  |  |
| **→2)-Man-(1→** | ^13^C | 101.46 | 79.03 |  |  |  |  |
| **B** | ^1^H | 5.33 | 4.10 |  |  |  |  |
| **→2)-Man-(1→** | ^13^C | 101.44 | 79.13 |  |  |  |  |
| **C** | ^1^H | 5.30 | 4.11 |  |  |  |  |
| **→2)-Man-(1→** | ^13^C | 101.43 | 79.03 |  |  |  |  |
| **D** | ^1^H | 5.28 | 4.11 |  |  |  |  |
| **→2)-Man-(1→** | ^13^C | 101.43 | 79.03 |  |  |  |  |
| **E** | ^1^H | 5.27 | 4.08 |  |  |  |  |
| **→2)-Man-(1→** | ^13^C | 101.43 | 79.24 |  |  |  |  |
| **F** | ^1^H | 5.13 | 4.08 |  |  |  |  |
| **Man-(1→** | ^13^C | 103.06 | 70.78 |  |  |  |  |
| **G** | ^1^H | 5.12 | 3.93 | 3.97 | 3.84 | 4.17 | 1.24 |
| **→3)-α-Fuc-(1→** | ^13^C | 102.18 | 68.53 | 78.26 | 71.29 | 67.97 | 16.45 |
| **H** | ^1^H | 5.09 | 3.81 |  |  |  |  |
|  | ^13^C | 102.26 | 69.30 |  |  |  |  |
| **I** | ^1^H | 5.07 | 4.07 |  |  |  |  |
|  | ^13^C | 102.97 | 69.25 |  |  |  |  |
| **J** | ^1^H | 5.05 | 3.84 |  |  |  |  |
|  | ^13^C | 98.80 | 78.62 |  |  |  |  |
| **K** | ^1^H | 5.01 | 3.85 |  |  |  |  |
|  | ^13^C | 98.91 | 78.52 |  |  |  |  |

**Table C.** ^1^H and ^13^C chemical shift assignments of the disaccharides obtained from partial hydrolysis of Tramesan. Signals are referred to acetone (2.225 ppm for ^1^H and 31.07 ppm for ^13^C).

| **Residues** | **Nucleus** | **Chemical shifts (ppm)** | | | | | |
| --- | --- | --- | --- | --- | --- | --- | --- |
|  |  | **1** | **2** | **3** | **4** | **5** | **6** |
| **A** | ^1^H | 5.26 | 3.80 | 3.78 | 4.03 | 4.26 | 3.69-3.84 |
| **→6)-α-Gal** | ^13^C | 93.31 | 71.68 |  | 70.29 | 69.35 | 67.24 |
| **B** | ^1^H | 5.21 | 3.88 | 3.95 | 3.86 | 4.22 | 1.18 |
| **→3)-α-Fuc** | ^13^C | 93.11 | 70.85 | 79.91 | 70.24 | 67.19 | 16.22 |
| **C** | ^1^H | 5.09 | 4.08 | 3.90 | 3.66 | 3.77 |  |
| **Man-(1→** | ^13^C | 103.21 | 70.94 | 70.70 | 67.74 | 74.12 |  |
| **D** | ^1^H | 4.97 | 3.82 | 3.86 | 3.97 |  |  |
| **α-Gal-(1→** | ^13^C | 99.49 | 69.21 | 70.24 | 71.82 |  |  |
| **E** | ^1^H | 4.60 | 3.56 | 3.73 | 3.90 | 3.81 | 1.22 |
| **→3)-β-Fuc** | ^13^C | 97.12 | 71.88 | 81.57 | 71.55 | 71.68 | 16.22 |
| **F** | ^1^H | 4.59 | 3.50 | 3.65 | 3.98 |  | 3.69-3.84 |
| **→6)-β-Gal** | ^13^C | 97.19 | 72.77 | 73.56 | 69.91 |  | 67.24 |


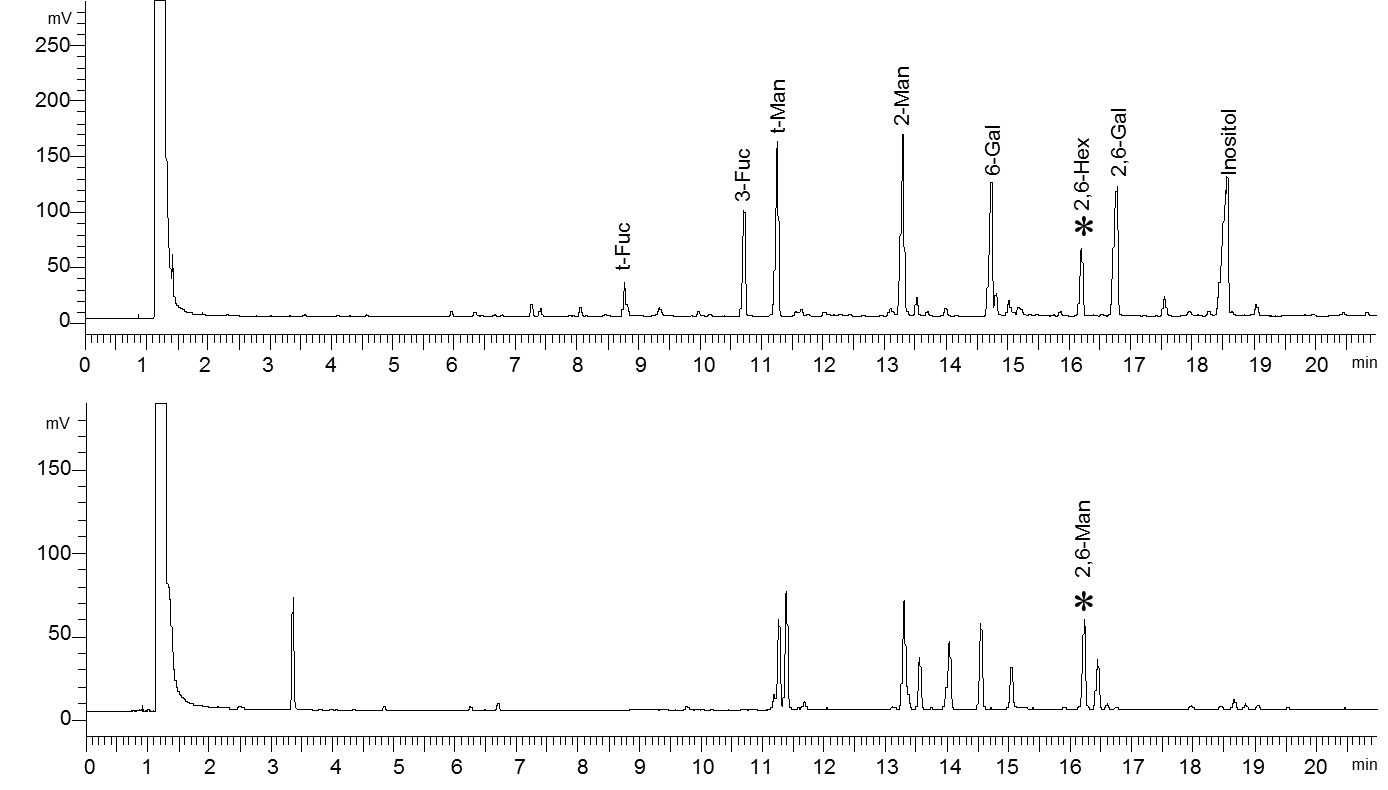


**Figure H.** Comparison of GLC elution profiles between PMAA derivatives of Tramesan and a sample containing 2,6-Man. The peaks of interest are marked with asterisks.
